# Multi-Analytical Characterization of the Earthen Plaster and Ground Layer from the Nirvana Platform in Cave No. 47 of the Kizil Grottoes, Xinjiang, China

**DOI:** 10.3390/ma19184024

**Published:** 2026-09-21

**Authors:** Liping Xue, Ling Shen, Jie Yang, Zhibo Zhou

**Affiliations:** 1 Department of Archaeology, Hangzhou City University, Hangzhou 310015, China; xueliping@hzcu.edu.cn (L.X.); shenling@hzcu.edu.cn (L.S.); 2 College of Engineering and Technology, China University of Geosciences (Beijing), Beijing 100083, China; yang_jie19871021@163.com; 3 The Qiuci Academy of Xinjiang, Urumqi 830000, China; 4 Key Laboratory of Cultural Heritage Interpretation & Technology for the Western Regions Governance, Ministry of Culture and Tourism, Urumqi 830000, China; 5 School of Cultural Heritage, Northwest University, Xi’an 710069, China

**Keywords:** Kizil Grottoes, Nirvana platform, stratigraphic reconstruction, earthen plaster, ground layer, anhydrite, hematite, animal hair

## Abstract

**Highlights:**

- Reconstructs a four-layered earthen plaster and ground layer sequence at Kizil Cave No. 47.
- Reveals layer-specific use of barley-processing waste in coarse clay layer and animal hair in fine clay layer.
- Suggests the possible presence of an ovalbumin-containing material within the earthen plaster based on ELISA.

**Abstract:**

The Nirvana platform in Cave No. 47 of the Kizil Grottoes preserves an important example of early Buddhist earthen plaster and ground layer manufacture along the Silk Roads. This study investigated the material composition and stratigraphic sequence of its earthen plaster and ground layer using optical microscopy (OM), micro-Raman spectroscopy, Scanning Electron Microscopy and Energy Dispersive X-ray Spectroscopy (SEM-EDX), X-ray diffraction (XRD), micro-Fourier transform infrared spectroscopy (µ-FTIR), laser-diffraction particle-size analysis (LD-PSA), indirect enzyme-linked immunosorbent assay (ELISA), and archaeobotanical observation. The results reveal a differentiated multilayer system consisting of a coarse clay layer, a fine clay layer, an anhydrite-dominated white ground layer, and a hematite-bearing, anhydrite-rich red ground layer. The coarse clay layer exhibited a broader particle-size distribution and contained abundant chopped straw and barley husks. The fine clay layer was more homogeneous and contained numerous keratinous animal hairs, compatible with a provisional attribution to cattle. A weakly positive ELISA response suggests the possible presence of an ovalbumin-containing material within the earthen plaster. The results demonstrate systematic differentiation of mineral fractions and organic inclusions across successive layers. The identified sequence reflects local technological choices within broader Asian traditions and provides a material basis for understanding and conserving the Nirvana platform.

## 1. Introduction

**Figure 1:**
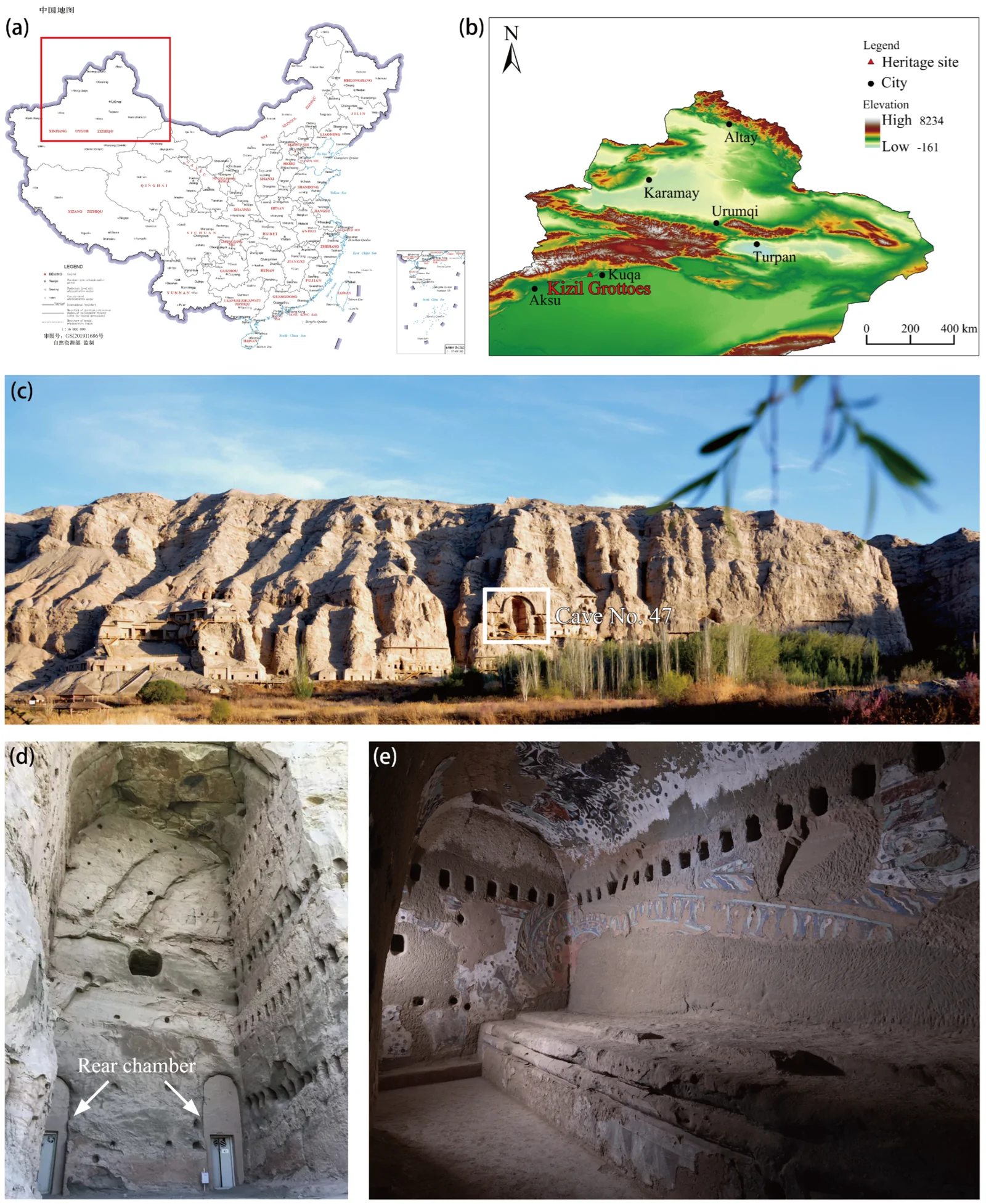
Location and context of Kizil Cave No. 47. (**a**) The map of China and the location of the research area (the base map image was sourced from http://bzdt.ch.mnr.gov.cn/, map approval No. GS(2019)1686, accessed on 17 July 2026). (**b**) Location of the Kizil Grottoes. (**c**) Exterior view of the Kizil Grottoes and the location of Cave No. 47. (**d**) Entrance and rear chamber position. (**e**) Rear chamber and surviving Nirvana platform.

The Kizil Grottoes are located on the northern margin of the Tarim Basin in present-day Xinjiang, China, within the historical oasis region of Qiuci (Kucha) (Figure 1a,b). Situated at an important crossroads of the ancient Silk Roads, the site preserves extensive evidence for the development and transmission of Buddhist architecture, image-making traditions, pigments, organic materials, and related technologies across Eurasia [1,2,3,4,5,6,7]. The Kizil Grottoes constitute the earliest Buddhist cave complexes in China, comprising hundreds of caves and a substantial corpus of mural paintings. In 2014, the site was inscribed on the UNESCO World Heritage List as part of the Silk Roads: the Routes Network of Chang’an–Tianshan Corridor.

Among the architectural forms represented at the Kizil Grottoes, the Giant Buddha caves, traditionally termed Da Xiang Ku, are distinguished by their exceptionally tall interiors and their association with monumental Buddha statues. Seven caves of this type have been identified at Kizil and represent an important component of the Buddhist architectural and artistic traditions of ancient Kucha [4]. Cave No. 47 of the Kizil Grottoes is the largest and one of the most representative examples (Figure 1c), reaching approximately 16.7 m at its highest point. Generally dated to the 4th century CE, it is regarded as the earliest large-scale Giant Buddha cave in the world and occupies an important position in the development of monumental Buddhist cave architecture [3,8,9]. Cave No. 47 comprises a lofty main chamber (Figure 1d) that originally accommodated a standing Buddha and a rear chamber containing a Nirvana platform (Figure 1e). The standing Buddha, which formed the principal focus of worship in the main chamber, is no longer preserved. The rear chamber and its platform constituted a ritual space associated with the Buddha’s parinirvana and would originally have supported a monumental reclining Buddha image.

The early date and scale of Cave No. 47 make it an important comparative case for studying Giant Buddha caves across Central and East Asia. In particular, Cave No. 47 has frequently been discussed in relation to the colossal Buddhas of Bamiyan (Afghanistan). Although the two sites developed in different historical and artistic contexts, both employed earthen materials, fibrous additives, a white ground layer, and polychrome painting in the construction and completion of monumental images [10,11,12,13,14,15]. Because Cave No. 47 may predate the surviving Buddhas at Bamiyan, it provides an important point of reference for examining the transmission, adaptation, and diversification of monumental Buddhist image-making technologies along the Silk Roads.

Scientific investigations of the Kizil Grottoes have increased in recent years, identifying inorganic pigments, metallic-foil decoration, animal glue, and drying oils [16,17,18,19,20]. However, most analytical studies have concentrated on painted and gilded surfaces, whereas the underlying earthen plaster and ground layer remain insufficiently documented. Their composition and stratigraphic relationships are important because they influence shrinkage, cracking, moisture and salt transport, interlayer adhesion, and long-term deterioration.

Earthen murals and clay sculptures in north-western China commonly employ multilayer support systems. The coarse clay layer generally contains larger mineral particles and chopped plant fibres, whereas the thinner fine clay layer is prepared from more finely graded earthen materials and finer reinforcing inclusions [21]. Comparable strategies have been reported at Ajanta (India), Bamiyan, and other Asian cave sites, although the mineral components, fibres, binding materials, and layer sequences vary by region and period [22,23,24,25,26]. At Bamiyan, investigations of sculptural and mural fragments have identified straw, chaff, animal hair, quartz-rich fillers, proteinaceous materials, plant gums, dairy products, egg, and drying oils in different modelling, sizing, ground, and paint layers [11,12,13,14,15]. The use of different fibrous materials in separate layers suggests that these additives may have been selected according to the intended role of each layer rather than incorporated as undifferentiated temper. However, several questions remain unresolved concerning the construction of multilayer earthen plasters, particularly whether differences in particle size, mineral composition, and organic inclusions reflect deliberate functional differentiation between layers or simply variability in available raw materials and preparation practices. The technological roles of plant- and animal-derived inclusions, and the extent to which such choices were consistently associated with particular stratigraphic layers, also remain incompletely understood. Before the present study, however, it had not been analytically established whether the earthen plaster and ground layer of the Nirvana platform in Cave No. 47 exhibited a comparable differentiation in mineral composition, particle-size distribution, fibrous additives, and organic materials. Importantly, the upper halo area of the Nirvana platform investigated in this study was executed as a clay relief (duisu), rather than as a purely planar painted surface. Comparable relief elements are preserved in several caves of the Kizil Grottoes, particularly in the halos associated with Buddha images. Despite their visual and technical importance, the material composition and manufacturing technology of these relief elements have received little dedicated analytical investigation.

In this study, samples from the Nirvana platform in the rear chamber of Cave No. 47 of the Kizil Grottoes were investigated using a multi-analytical approach. The principal aim was to characterize the constituent materials and stratigraphic sequence of the earthen plaster and ground layer, with particular attention to possible differentiation in particle-size distribution, mineral composition, fibrous inclusions, and proteinaceous materials between successive layers. By clarifying how these components were distributed within the multilayer system, the study seeks to improve understanding of the material choices and construction practices employed in the Nirvana platform and to provide an analytical basis for comparison with related Buddhist earthen plaster and ground layer technologies documented at broadly contemporaneous and later cave sites in other regions. The results also provide material information relevant to the interpretation and conservation of the surviving reliefs and painted surfaces in the Kizil Grottoes.

## 2. Materials and Methods

### 2.1. Sampling Materials

**Figure 2:**
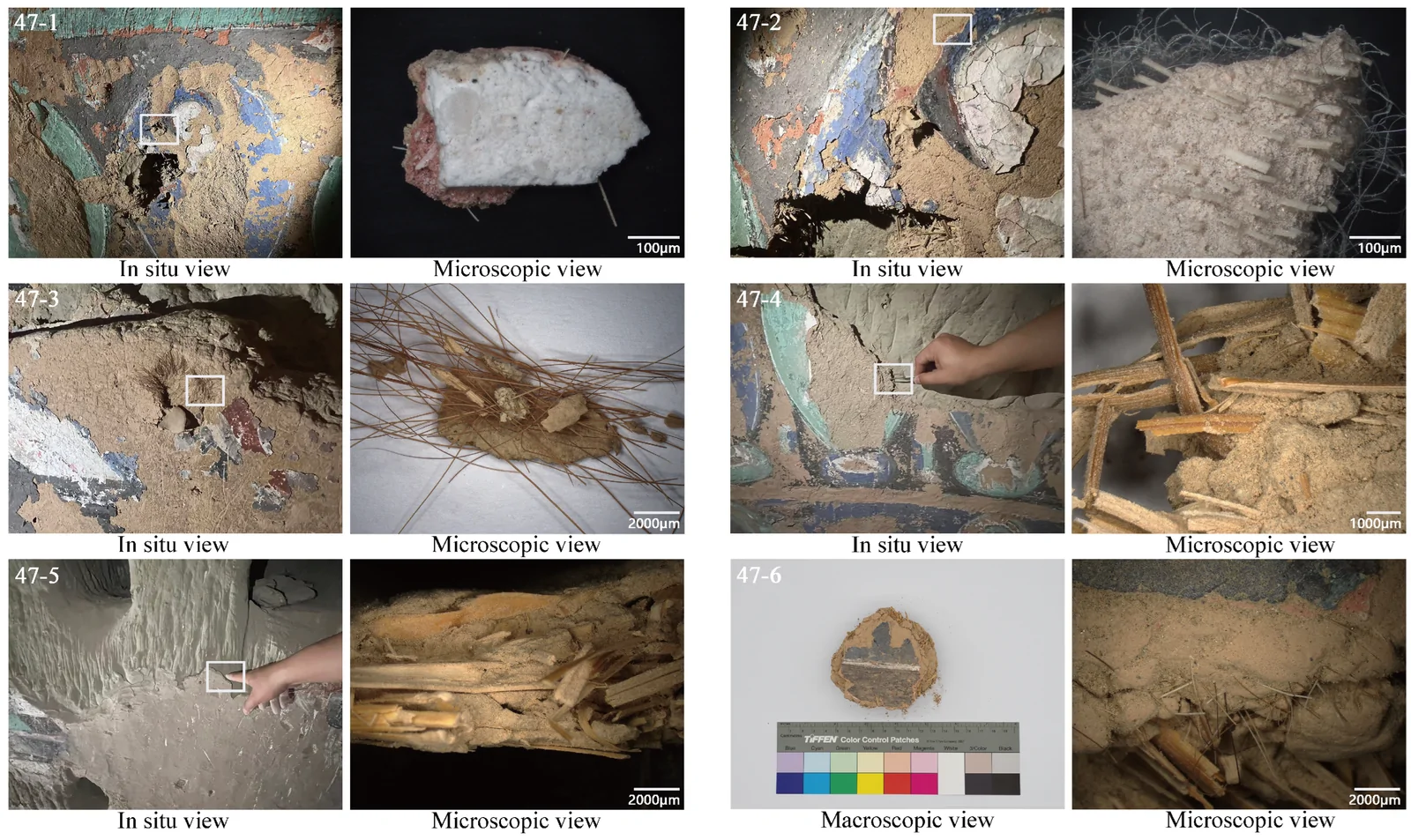
Representative in situ and microscopic views of the investigated samples. White rectangles indicate the sampling locations.

Six samples were selected for analysis, representing the white ground layer, clay layer, and a detached multilayer fragment (Table 1, Figure 2). Samples 47-1 to 47-4 were collected from the upper halo area of the Nirvana platform, whereas sample 47-5 was obtained from the right wall of the rear chamber. Sample 47-6 was taken from a detached fragment found on the Nirvana platform, and its original location could not be determined. Sampling was restricted to already detached, unstable, or damaged areas and was undertaken with the minimum quantity required for analysis. Analytical methods were selected according to the material represented by each sample and its suitability for the corresponding method. Because only limited quantities of archaeological material were available, not all samples were subjected to the same analytical procedures. Sample 47-1 was selected for Raman and SEM-EDX characterization of the ground layers. Representative animal hairs from samples 47-3 and 47-6 were examined by SEM, while animal fibres from samples 47-1 and 47-2 were analysed by µ-FTIR. Sample 47-6, which preserved the most complete multilayer sequence and separable coarse and fine clay layers, was additionally used for XRD, LD-PSA, and archaeobotanical examination. Samples 47-4 and 47-5 were screened for proteinaceous materials by ELISA.

### 2.2. Optical Microscopy (OM)

The basic characteristics of samples were observed under optical microscopy using a Soptop RX50M metallurgical microscope (Ningbo Sunny Optical Instrument Co., Ltd., Ningbo, China) or a Keyence VHX-7000 digital microscope (KEYENCE Corporation, Osaka, Japan), depending on the dimensions of the samples. Observations focused on layer sequence, texture, particle grading, fibre morphology and plant inclusions.

### 2.3. Micro-Raman Spectroscopy

Micro-Raman analysis was performed using a Renishaw inVia Reflex micro-Raman spectrometer (Renishaw plc, Gloucestershire, UK) equipped with an electron-multiplying charge-coupled device (EM-CCD) detector. A 785 nm laser was employed to characterize the mineral composition of the white and red ground layers. Spectra were collected over the Raman-shift range of 100–1300 cm^−1^ using a laser power of 0.1–2 mW. The acquisition time was set to 2–10 s, and each measurement comprised 3–5 accumulations. Fluorescence-background removal and mild smoothing were applied only for visualization, and mineral phases were identified qualitatively by comparing the acquired spectra with reference spectra and published data.

### 2.4. Scanning Electron Microscopy and Energy Dispersive X-Ray Spectroscopy (SEM-EDX)

SEM-EDX examination was carried out using a Phenom XL G2 scanning electron microscope (Thermo Fisher Scientific, Eindhoven, The Netherlands) equipped with secondary electron (SE), backscattered electron (BSE), and energy-dispersive X-ray (EDX) detectors. Animal hairs isolated from samples 47-3 and 47-6 were coated and observed under high vacuum (0.10 Pa) using secondary-electron imaging at 10 kV. A fragment of sample 47-1 was embedded in epoxy resin and polished to expose the stratigraphic cross-section. The uncoated cross-section was examined in low-vacuum mode (60 Pa) using backscattered-electron imaging at 15 kV.

### 2.5. X-Ray Diffraction (XRD)

The crystalline mineral phases of the fine and coarse clay plasters of sample 47-6 were analysed using a SmartLab 9 kW diffractometer (Rigaku Corporation, Tokyo, Japan). The measurements were performed at room temperature using Cu Kα radiation (λ = 1.54059 Å), with the generator operated at 45 kV and 100 mA. Diffraction patterns were recorded over a 2θ range of 5–80° at a scanning rate of 2° min^−1^. A nickel filter was used to suppress Cu Kβ radiation. Prior to measurement, the diffractometer was calibrated using a silicon standard. The crystalline phases were identified using JADE 6.5 software by comparison with reference patterns in the International Centre for Diffraction Data Powder Diffraction File (PDF) database. Samples from the fine and coarse clay layers were analysed separately to determine differences in their mineralogical compositions.

### 2.6. Micro-Fourier Transform Infrared Spectroscopy (µ-FTIR)

Animal fibres from samples 47-1 and 47-2 were examined using a Thermo Scientific™ Nicolet™ iN10 FTIR microscope (Thermo Fisher Scientific, Waltham, MA, USA). Individual fibres were placed in a diamond compression cell and gently compressed only to reduce specimen thickness and obtain adequate infrared transmission, while avoiding excessive deformation of the fibres. The analysis was conducted to characterize selected animal-hair fibres and to assess their proteinaceous and keratinous nature. Spectra were acquired over the range of 4000–400 cm^−1^ at a resolution of 4 cm^−1^ with 64 scans for each measurement. The spectra were stored in transmittance units and evaluated by comparison with the characteristic absorption bands of keratinous materials and published reference data.

### 2.7. Laser-Diffraction Particle-Size Analysis (LD-PSA)

Particle-size distributions of the fine and coarse clay layers from sample 47-6 were determined by wet laser diffraction using a Malvern Mastersizer 2000 particle-size analyzer (Malvern Instruments Ltd., Malvern, UK) equipped with a Hydro 2000SM (A) wet-dispersion unit. The samples were dispersed in water, for which a refractive index of 1.330 was used. Measurements were performed over a particle-size range of 0.020–2000 μm, and the results were expressed as volume-based particle-size distributions and volume-equivalent spherical diameters. Data were calculated using the single-narrow-mode spherical-particle model with enhanced sensitivity and a particle absorption index of 0.1. Three consecutive measurements were conducted for each sample, with obscuration values of 5.03–5.10% for the fine layer and 9.30–9.35% for the coarse layer. The particle-size distribution curves represent the mean distributions obtained from the three measurements. For each individual measurement, the volume-weighted mean diameter D[4,3], surface-weighted mean diameter D[3,2], d(0.1), d(0.5), d(0.9), span, and specific surface area were recorded. These derived parameters are reported as the mean ± standard deviation (SD) of the three measurements.

### 2.8. Indirect Enzyme-Linked Immunosorbent Assay (ELISA)

Samples 47-4 and 47-5 were screened for ovalbumin, casein, and type I collagen by indirect ELISA. Samples were handled separately using clean disposable consumables to minimize the risk of cross-contamination during preparation and analysis. Crushed samples were extracted in 1 mL carbonate–bicarbonate coating buffer (0.05 mol L^−1^, pH 9.6) with 30 min ultrasonication, and allowed to settle. The resulting supernatants were transferred to 96-well microplates at 60 μL per well, with three replicate wells prepared for each sample. Coating-buffer blanks and appropriate positive controls were included and incubated with the samples at 4 °C overnight. After blocking with 1% bovine serum albumin in phosphate-buffered saline (PBS, pH 7.6) at 37 °C for 1 h, the wells were incubated separately with anti-ovalbumin (C6534, Sigma-Aldrich, St. Louis, MO, USA), anti-casein (ab166596, Abcam, Cambridge, UK), or anti-type I collagen antibodies (14695-1-AP, Proteintech Group, Rosemont, IL, USA) for 1 h at 37 °C. An HRP-conjugated Affinipure goat anti-rabbit IgG (H + L) secondary antibody (Proteintech Group, Rosemont, IL, USA) was then applied after washing the wells three times with TBST. Colour development was performed using 3,3′,5,5′-tetramethylbenzidine (TMB) for 5 min and terminated with 2 mol L^−1^ H_2_SO_4_. Absorbance was measured at 450 and 630 nm using an Epoch microplate spectrophotometer (BioTek Instruments, Winooski, VT, USA). The corrected absorbance was calculated as A = A_450_ − A_630_, and the limit of detection (LOD) was calculated from six blank wells as the mean blank absorbance plus three standard deviations.

### 2.9. Archaeobotanical Observation

Cereal-like plant remains were manually separated from the coarse clay layer of sample 47-6 and examined under a Keyence VHX-7000 digital microscope. Identification was based on overall shape, longitudinal furrow and husk morphology.

## 3. Results

### 3.1. Sample Stratigraphy and Layer Characteristics

Microscopic examination revealed clear differences in the stratigraphy and incorporated materials of the samples. Sample 47-1 preserved the upper portion of the fine clay layer together with the overlying white and red ground layers. A small number of animal hairs were observed at or immediately below the interface between the fine clay layer and the ground layer. Sample 47-2 was a hair-bearing tissue fragment of animal hide recovered from the fine clay layer and sample 47-3 consisted of a fine clay layer containing abundant animal hairs. Samples 47-4 and 47-5 were both derived from the earthen plaster and contained numerous straw-like plant stems. Representative microscopic views of samples 47-1–47-5 are provided in Figure 2.

**Figure 3:**
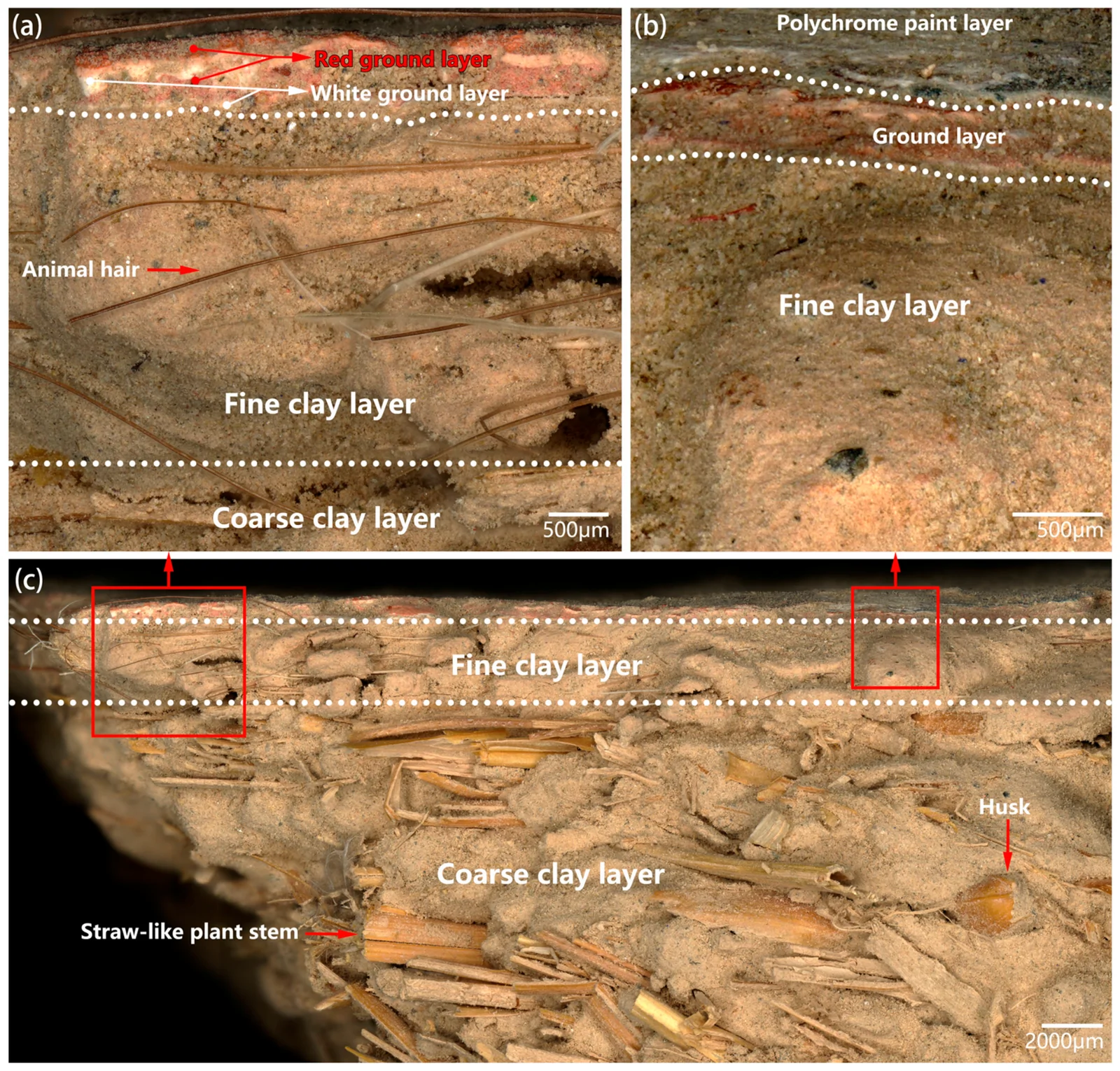
Optical-microscopic stratigraphy of detached multilayer sample 47-6. (**a**,**b**) Enlarged views of the selected areas. (**c**) Overall cross-sectional view of the fragment.

Sample 47-6 was a detached multilayer fragment preserving the most complete stratigraphic sequence (Figure 3) among the analysed samples. A relatively thick coarse clay layer could be clearly distinguished from the thinner and more homogeneous fine clay layer above it. The coarse clay layer comprised a heterogeneous mineral matrix containing abundant straw-like plant stems and husks (Figure 3c), whereas the fine clay layer contained fewer coarse particles and numerous slender animal hairs (Figure 3a). Above the fine clay layer, the white and red ground layers formed thin, continuous bands. Their boundaries were locally irregular and interwoven rather than uniformly planar, and patches of the two ground materials penetrated one another at the sub-millimetre scale. In the better-preserved areas, a paint layer coating the ground layer was also observed (Figure 3b).

Taken together, the samples indicate a representative stratigraphic sequence consisting of an underlying support, either the rock substrate or sculptural core, followed by the coarse clay layer, fine clay layer, white ground layer, red ground layer, and one or more polychrome paint layers. The coarse and fine clay layers constitute the earthen plaster, whereas the white and red layers constitute the ground layer system.

### 3.2. Mineralogical and Elemental Characterization of the Ground Layer

**Figure 4:**
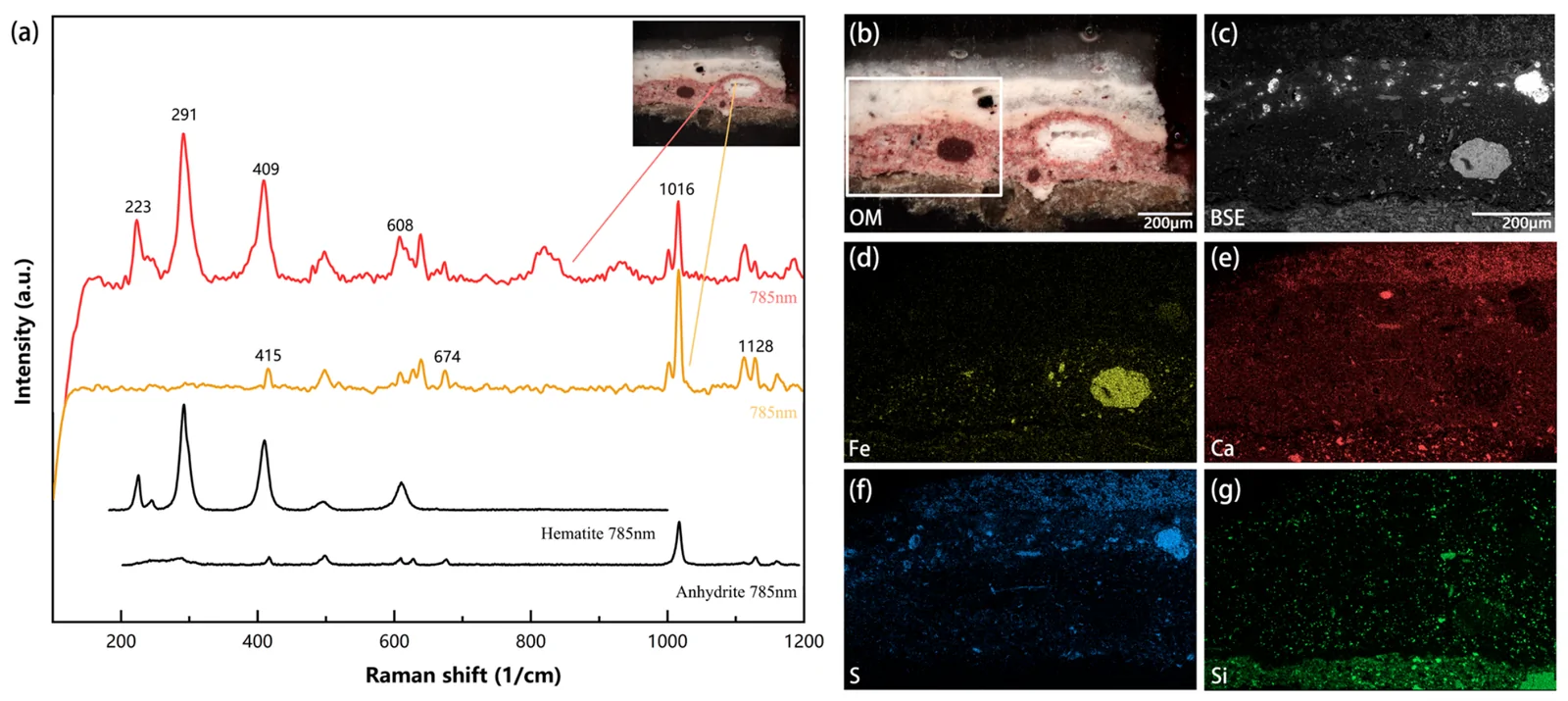
Mineralogical and elemental characterization of the ground layer in embedded sample 47-1. (**a**) Raman spectra of the white ground layer (yellow) and pale red ground layer (red), compared with anhydrite (RRUFF ID R061102) and hematite (RRUFF ID R060190) reference spectra from the RRUFF online database (https://rruff.info/ (accessed on 28 July 2026)). (**b**) Optical-microscopic image of the polished cross-section, the white box indicates the area shown in the BSE image in (**c**). (**c**) BSE image of the analysed area of the cross-section, and the corresponding elemental maps of (**d**) Fe, (**e**) Ca, (**f**) S, and (**g**) Si.

The Raman spectrum acquired from the white ground layer of sample 47-1 (Figure 4a) exhibited a strong band at approximately 1016 cm^−1^, accompanied by bands at approximately 416, 499, 610, 627, 674, 1111, 1128, and 1162 cm^−1^. The intense peak at 1016 cm^−1^ was assigned to the symmetric stretching vibration (ν_1_) of SO_4_^2−^. The bands at 1111, 1128, and 1162 cm^−1^ were attributed to asymmetric sulfate stretching vibrations (ν_3_), whereas those at 610, 627, and 674 cm^−1^ were associated with sulfate bending vibrations (ν_4_). The lower-wavenumber bands near 416 and 499 cm^−1^ were assigned to ν_2_ bending modes. Taken together, this band pattern closely matches reported Raman spectra of anhydrite [27,28,29,30,31] and differs from gypsum, whose principal ν_1_ sulfate band typically occurs at approximately 1008 cm^−1^, with corresponding shifts in the sulfate stretching and bending modes. Anhydrite was therefore identified as the principal crystalline phase currently preserved in the white ground layer.

The Raman spectrum of the pale red ground layer (Figure 4a) was characterized by prominent bands at approximately 223, 291, 409, and 498 cm^−1^, together with a band in the 607–608 cm^−1^ region. The bands at approximately 223 and 498 cm^−1^ were assigned to the A_1_g lattice modes of hematite (α-Fe_2_O_3_), whereas those at approximately 291 and 409 cm^−1^ were assigned to E_g modes. These bands collectively provide diagnostic evidence for hematite [32,33,34,35,36,37]. The band near 608 cm^−1^ occurs in a spectral region where the E_g mode of hematite and the ν_4_ sulfate bending vibrations of anhydrite may overlap and was therefore not assigned exclusively to hematite. The presence of anhydrite was further supported by the strong band at approximately 1016 cm^−1^, assigned to the ν_1_ symmetric stretching vibration of SO_4_^2−^, together with bands at approximately 1113 and 1128 cm^−1^ attributable to ν_3_ asymmetric sulfate stretching and a weaker feature near 674 cm^−1^ in the ν_4_ sulfate bending region. The pale red ground layer is therefore interpreted as a hematite-bearing, anhydrite-rich ground material rather than a pure iron oxide layer.

The SEM-EDX results provided complementary evidence for the compositional differentiation of the stratigraphic layers (Figure 4b–g). In the BSE image (Figure 4c), the fine clay layer and the overlying ground layer could be distinguished by differences in texture and electron-density contrast. The Fe signal was enhanced in the pale red ground layer, with discrete Fe-rich particles also evident (Figure 4d), consistent with the Raman identification of hematite. Ca and S were both enriched within the ground-layer system and exhibited broadly overlapping spatial distributions, particularly in the white ground layer and parts of the pale red ground layer (Figure 4e,f). This spatial association is consistent with the Raman identification of anhydrite in both ground layers. In contrast, Si was more strongly distributed within the underlying fine clay layer (Figure 4g), reflecting the greater abundance of silicate-rich mineral components in the earthen plaster. Because EDX provides elemental rather than crystallographic information, the Ca–S association was interpreted in conjunction with the Raman identification of anhydrite rather than being used independently for phase assignment.

### 3.3. SEM Morphology and Micro-FTIR Identification of Animal Hair

**Figure 5:**
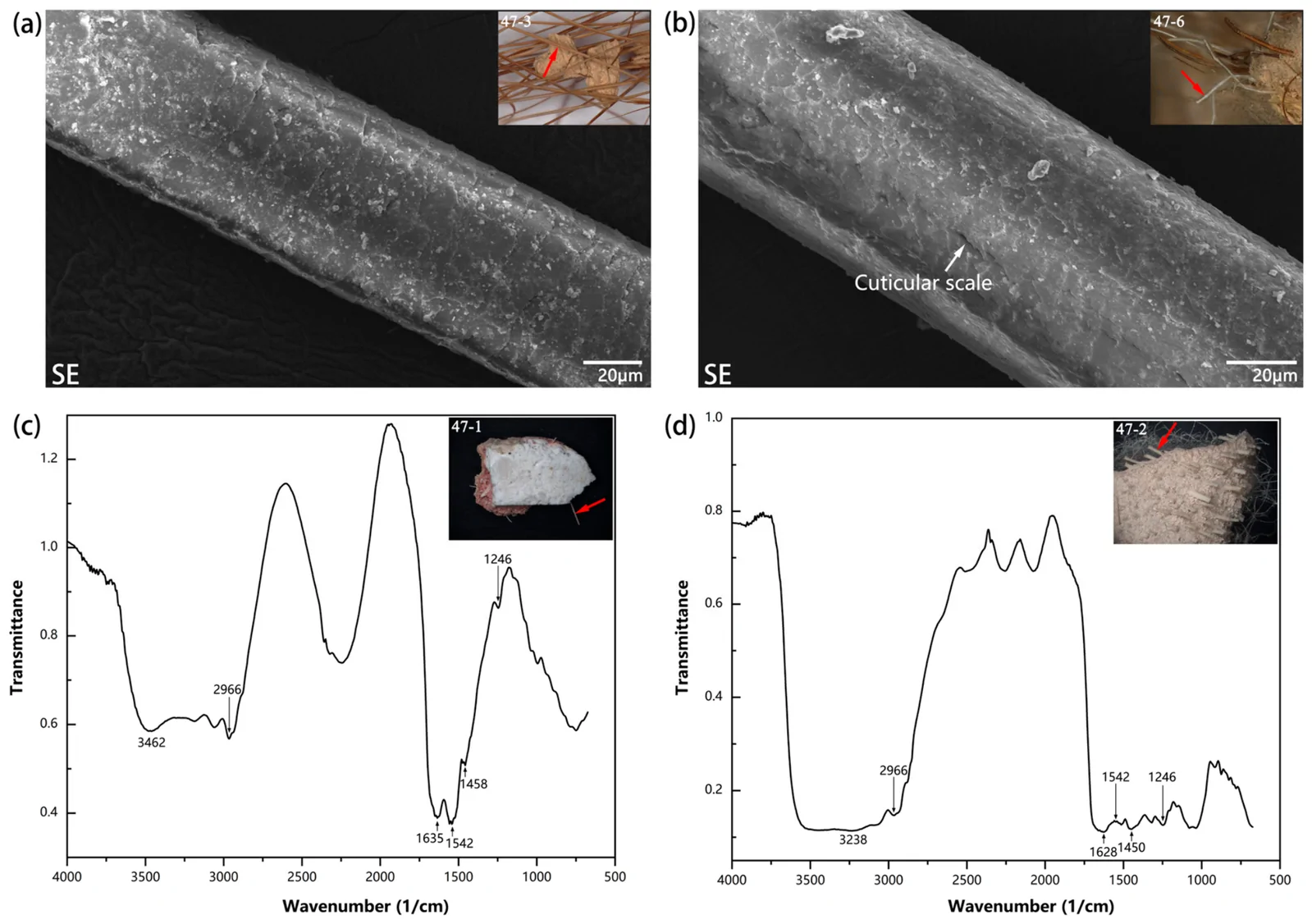
Morphological and FTIR characterization of animal hairs. (**a**) SE image of a representative animal hair from sample 47-3. (**b**) SE image of a representative animal hair from sample 47-6. (**c**) FTIR spectrum of an animal hair from sample 47-1. (**d**) FTIR spectrum of animal hair from sample 47-2. The red arrows indicate the specific analysed hairs.

Secondary-electron images of two hairs isolated from samples 47-3 and 47-6 showed cylindrical shafts bearing overlapping, flattened cuticular scales (Figure 5a,b). The scales formed a predominantly imbricate to mosaic-like arrangement with irregular to wavy margins. Mineral particles adhered extensively to the surfaces, and local lifting, cracking, and loss of cuticular scales were observed. Comparable imbricate or mosaic-like cuticular patterns have been reported in bovine hair, whereas imbricate patterns also occur in goat and horse hair [38,39,40,41]. Accordingly, the SEM morphology supports identification as mammalian hair and is compatible with cattle hair, but the observed cuticular pattern alone is not species-diagnostic [42].

The FTIR spectra of the animal hairs from samples 47-1 and 47-2 exhibited absorption features characteristic of keratinous proteins (Figure 5c,d). Bands were observed at approximately 3462/3238, 2966, 1635/1628, 1542, 1458/1450, and 1246 cm^−1^. The broad bands at 3462 and 3238 cm^−1^ were attributed mainly to N–H/O–H stretching in the amide A/O–H region, whereas the band at 2966 cm^−1^ was associated with aliphatic C–H stretching. The bands at 1635/1628 and 1542 cm^−1^ were assigned to amide I, dominated by C=O stretching, and amide II, arising mainly from N–H bending coupled with C–N stretching, respectively. The bands at 1458/1450 cm^−1^ were attributed to CH_2_/CH_3_ deformation vibrations, while the band at 1246 cm^−1^ was assigned to amide III, involving C–N stretching and N–H bending [43,44,45]. Despite differences in baseline shape and relative band intensity, both spectra exhibited the characteristic amide I–III pattern of keratinous proteins, supporting identification of the analysed fibres as animal hair. Combined with the optical and SEM observations, the FTIR results are compatible with a provisional attribution to cattle hair. However, the spectroscopic and morphological evidence alone is insufficient for definitive species-level identification, which would require comparison with vouchered reference hairs or biomolecular analysis.

### 3.4. ELISA Screening for Proteinaceous Additives

The ELISA results are summarized in Table 2. Comparable immunological approaches have previously been applied to the identification of proteinaceous materials in ancient murals and other Chinese painted cultural relics [46,47]. Sample 47-4 showed replicate absorbance values of 0.082, 0.073, and 0.086 for ovalbumin, with a mean absorbance of 0.0803, exceeding the corresponding LOD of 0.0539. The calculated RI was 1.51%, classifying the response as weakly positive. In contrast, the mean ovalbumin absorbance of sample 47-5 (0.0403) remained below the LOD. Both samples were negative for casein and collagen type I, with mean absorbance values below the respective LODs of 0.1315 and 0.0669. Thus, among the three protein targets examined, only sample 47-4 produced a detectable, although weak, response to ovalbumin. No corresponding ovalbumin response was observed in sample 47-5, probably indicating variation between the two analysed earthen plaster samples. Because antigen detection in cultural heritage samples may be influenced by ageing and interactions between proteinaceous materials and the surrounding inorganic matrix, the weakly positive response was interpreted qualitatively rather than as a direct indication of protein concentration [24]. Similarly, the negative responses indicate that the corresponding target proteins were not detected under the applied analytical conditions, rather than demonstrating their complete absence.

### 3.5. Particle-Size and Mineralogical Differentiation of the Earthen Plaster

PSA and XRD have previously been used in combination to characterize the textural and mineralogical properties of archaeological earthen plasters [25,48]. The results obtained in this study revealed clear differences between the fine and coarse clay layers in both particle-size distribution and crystalline mineral composition.

#### 3.5.1. Particle-Size Distribution

**Figure 6:**
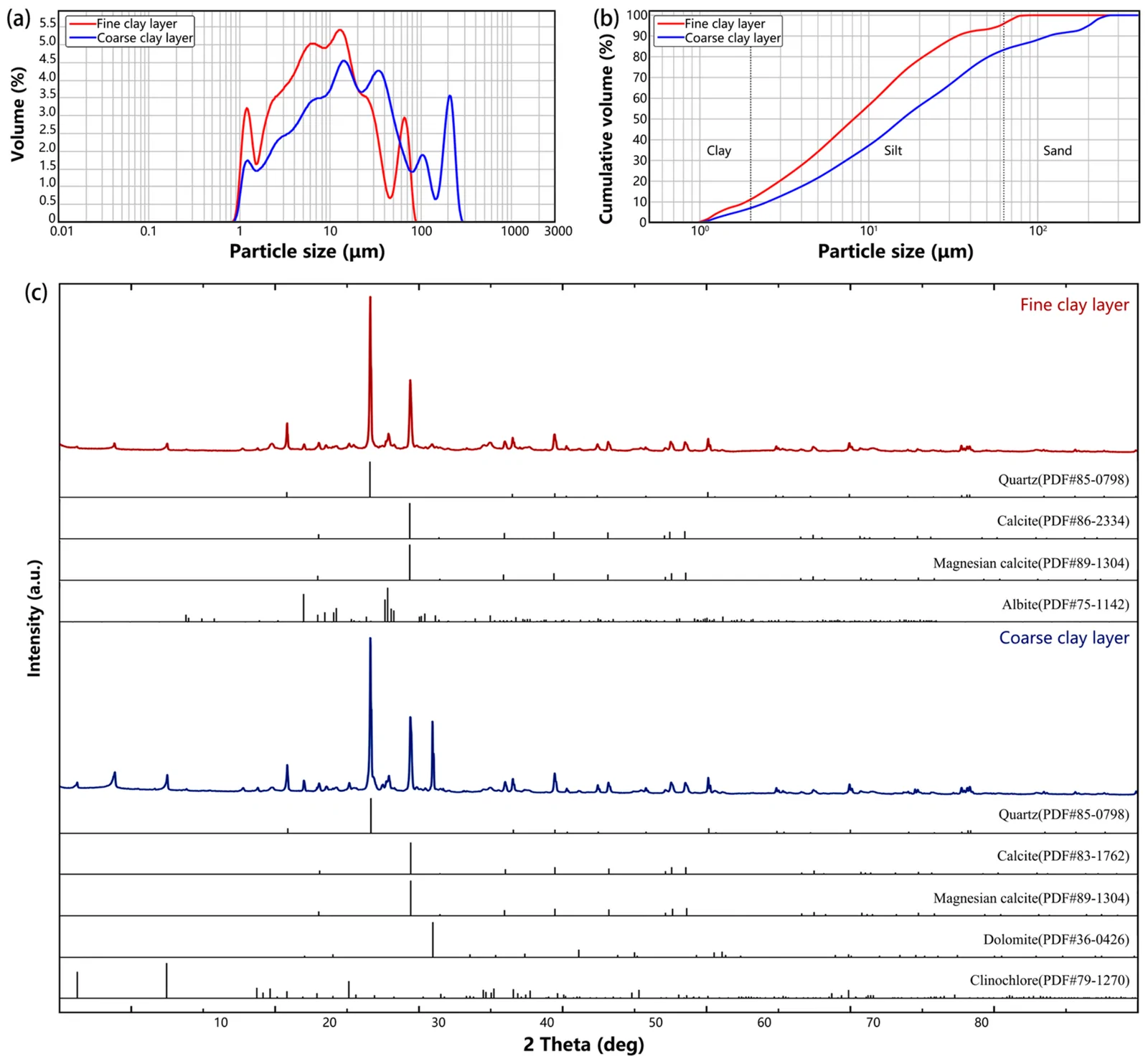
Granulometric and mineralogical characterization of sample 47-6. (**a**) Differential volume-based particle-size distributions. (**b**) Cumulative volume distributions. (**c**) XRD patterns.

The fine clay layer exhibited a narrower volume-based particle-size distribution that was shifted towards smaller particle sizes, whereas the coarse clay layer showed a broader distribution extending further into the coarse-particle range (Figure 6a,b). The fine clay layer had a volume-weighted mean diameter, D[4,3], of 14.291 ± 0.131 μm and a surface-weighted mean diameter, D[3,2], of 4.744 ± 0.035 μm. Its d(0.1), d(0.5), and d(0.9) values were 1.872 ± 0.009, 8.157 ± 0.096, and 33.415 ± 0.601 μm, respectively, with a span of 3.866 ± 0.027 and a specific surface area of 1.263 ± 0.012 m^2^/g. In comparison, the coarse clay layer had D[4,3] and D[3,2] values of 39.407 ± 2.170 and 7.014 ± 0.161 μm, respectively, together with d(0.1), d(0.5), and d(0.9) values of 2.509 ± 0.049, 15.817 ± 0.482, and 116.197 ± 10.287 μm, a span of 7.179 ± 0.434, and a specific surface area of 0.856 ± 0.020 m^2^/g (Table 3).

The difference between the two layers was therefore most pronounced in the upper part of the particle-size distribution. The d(0.9) of the coarse clay layer was approximately 3.5 times that of the fine clay layer, whereas the difference in d(0.1) was comparatively small. The cumulative distribution curves further showed that both layers consisted predominantly of silt-sized particles (Figure 6b); however, the coarse clay layer contained a substantially larger sand-sized fraction and retained measurable particles up to approximately 270 μm. By contrast, particles in the fine clay layer were concentrated mainly below approximately 80 μm. These results demonstrate purposeful or source-related textural differentiation between the fine and coarse clay layers. Comparable differentiation between superimposed coarse and fine earthen plaster layers has also been documented at other grotto sites in north-western China [21].

#### 3.5.2. Mineralogical Composition

The XRD patterns showed that the fine and coarse clay layers shared several crystalline mineral phases but also contained layer-specific components (Figure 6c). Quartz (SiO_2_), calcite (CaCO_3_), and magnesian calcite were identified in both layers. Albite (NaAlSi_3_O_8_) was additionally identified in the fine clay layer. In contrast, dolomite [CaMg(CO_3_)_2_] and clinochlore were identified in the coarse clay layer but were not identified in the fine clay layer. The two layers therefore possessed related silicate–carbonate mineral assemblages, while differing in the occurrence of feldspar, dolomite, and chlorite-group phases. The accessory differences may reflect natural heterogeneity, separate batches of raw earth, or deliberate selection and processing. Because the XRD analysis was qualitative, no quantitative comparison of mineral abundances was made from the relative peak intensities.

### 3.6. Identification of Cereal Plant Remains

**Figure 7:**
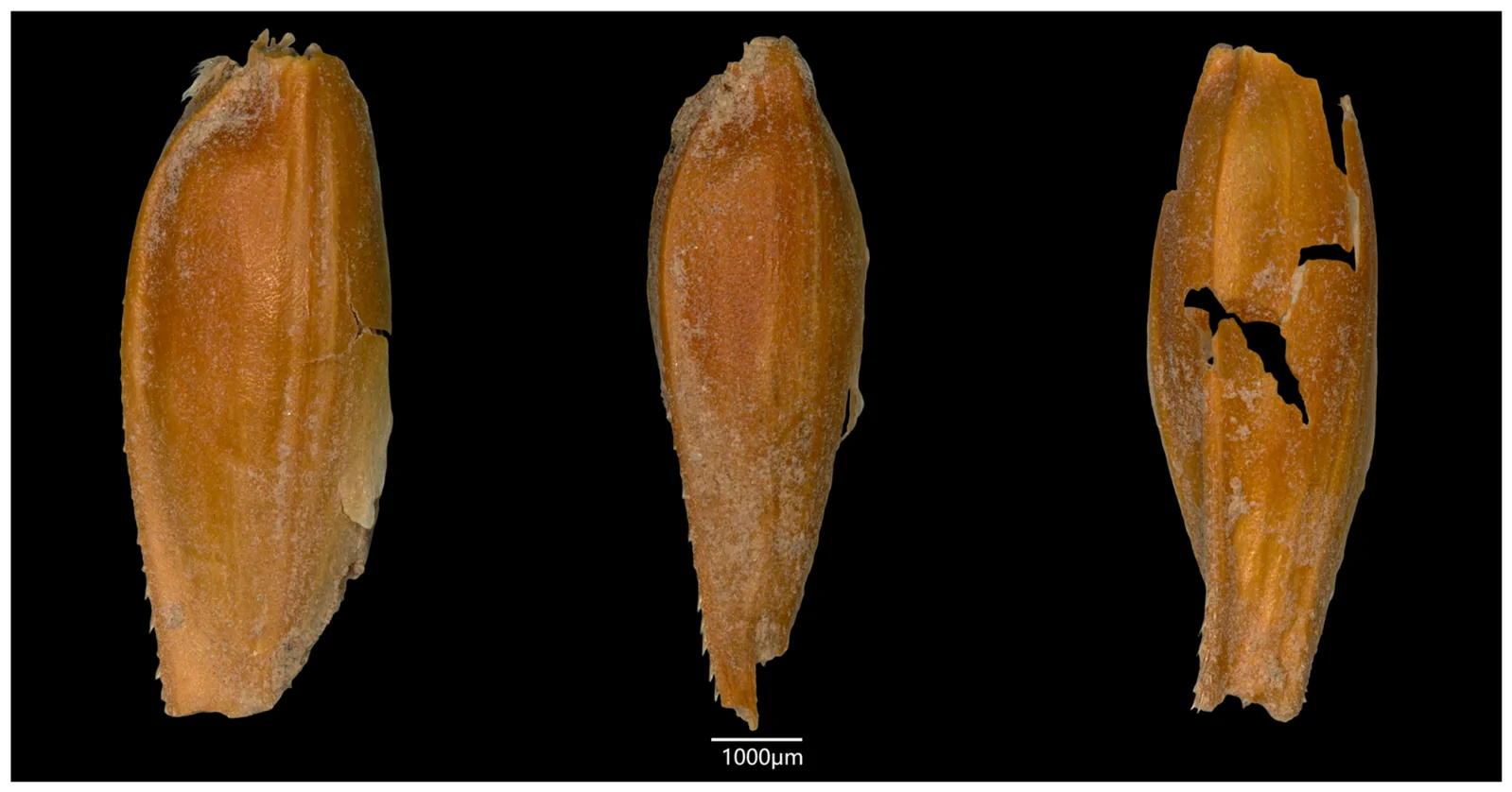
Three selected empty cereal husk remains from sample 47-6.

Three empty cereal husk remains were recovered from the coarse clay layer of sample 47-6 (Figure 7). No caryopses were preserved within the husks. The specimens exhibited elongated, spindle-shaped outlines, persistent enclosing bracts, prominent longitudinal ridges or furrows, and tapered ends. These morphological characteristics are consistent with the husks of hulled barley (*Hordeum vulgare* L.) described in archaeobotanical identification studies [49]. The preserved outlines showed a degree of lateral asymmetry, and preliminary examination by a plant specialist suggested a possible attribution to six-row barley.

Regional archaeobotanical evidence documents the cultivation of different barley forms in Xinjiang before the period represented by Cave No. 47 of the Kizil Grottoes. At Wupaer in the southwestern Tarim Basin, both two-rowed and six-rowed hulled barley were identified in deposits dating to approximately 1200–400 BCE [50]. In addition, barley assemblages from the eastern Tianshan dating from approximately 400 BCE to 300 CE have been interpreted as probably representing a six-row naked form [51]. These findings confirm that six-row barley was present in Xinjiang before the construction of Cave No. 47. Nevertheless, because the present specimens consist only of empty husks and lack preserved caryopses and diagnostic rachis segments, they were provisionally identified as hulled barley, possibly representing a six-row form. These husk remains therefore support the use of barley-processing waste in the coarse clay layer but do not by themselves prove that all observed stems derived from six-row barley.

## 4. Discussion

The combined stratigraphic and analytical results indicate that the earthen plaster and ground layer of the Nirvana platform were constructed as a deliberately differentiated multilayer system rather than as a homogeneous earthen coating. The terminology used here distinguishes two technological units: the earthen plaster comprises the coarse and fine clay layers, whereas the ground layer comprises the white and red ground layers. These four layers represent successive stages of preparation from the underlying support towards the paint layers. Comparable coarse-to-fine earthen sequences followed by mineral grounds have been documented in Buddhist mural paintings and clay sculptures in north-western China, Central Asia, and India [21,25,48,52,53,54,55,56,57]. The system identified in the Nirvana platform therefore belongs to a wider tradition, but it is distinguished by the coordinated differentiation of particle size, mineral composition, organic inclusions, and ground layer composition. The present investigation is also significant because several samples from the upper halo area relate to a relief rather than solely to a planar wall-painting support, providing an initial material reference for interpreting the construction of relief elements in the Kizil Grottoes. Given the conservation-driven restrictions on sampling, the reconstructed sequence should be regarded as representative of the analysed materials rather than necessarily of all areas of the Nirvana platform. The differentiated use of coarse and fine clay plasters, fibrous inclusions, and calcium sulfate-based grounds identified here may therefore offer a useful basis for future comparative investigations of reliefs at Kizil and other Kucha grottoes.

Particle-size analysis provides the clearest quantitative evidence for textural differentiation between the two clay layers. Their finest fractions were relatively similar, whereas the coarse clay layer contained a substantially broader upper particle-size range and a larger coarse fraction. The XRD results are consistent with this distinction: both layers contain quartz and carbonate phases but differ in several accessory minerals. Together, these observations are consistent with separate preparation, differential grading, or the use of different batches of raw earth. Screening, sedimentation, or selective use of sediment fractions could have produced the contrast. Experimental studies have shown that base and finishing earthen plasters can be prepared using differently sieved earths [58], although the specific procedure employed at the Nirvana platform cannot be determined from the present data. In technological terms, the broader-graded coarse mixture may have supplied the volume needed to cover or level an irregular support, whereas the thinner fine clay layer produced a more homogeneous surface beneath the white ground. Previous studies indicate that particle grading and fibrous inclusions can influence the drying and cracking behaviour of earthen materials [21,53,59]. The principal conclusion is that the craftsmen adjusted the texture and composition of each mixture according to its position within the earthen plaster. This differentiated preparation may have been intended to balance adequate adhesion to the underlying rock support with reduced drying shrinkage and cracking, while also providing a sufficiently smooth surface for the application of the ground layer.

The fibrous inclusions provide particularly strong evidence for layer-specific selection. Chopped straw and barley husks were concentrated in the coarse clay layer, whereas numerous slender animal hairs occurred in the fine clay layer. Plant fibres are widely documented as additives in historic earthen plasters [60,61,62]. In north-western Chinese grotto murals, double-layer plaster systems commonly comprised a straw-bearing coarse layer and a fine upper layer reinforced with finer fibres such as hemp or jute [21,53,63,64,65,66]. In the Nirvana platform of Cave No. 47, however, the corresponding upper fine clay layer appears to have been reinforced mainly with animal hair. Traditional Tibetan mural construction has been described as employing cattle hair in a finer coating layer, although animal hair was not observed in the two historic buildings at Gyantse [67]. Chopped straw or animal hair has also been reported in Central Asian Buddhist wall-painting preparations [52], and animal hair occurred in the mud coating of the Giant Buddhas at Bamiyan [68] and in Buddhist plasters from Gandhara [69]. The prominent use of animal hair in the fine clay layer at Cave No. 47 is therefore less common among the north-western Chinese grottoes but remains technologically plausible within the broader Central Asian tradition. Its use is compatible with the production of a relatively thin and uniform finishing layer, although its precise reinforcing effect remains to be tested. SEM morphology suggests that the fibres were most consistent with cattle hair, although this attribution remains provisional. FTIR spectra from hairs in other samples support theirkeratinous nature but does not distinguish cattle from sheep, goat, horse, or other mammals without a validated chemometric reference set. The combination of barley-processing waste and animal-derived fibres is nevertheless consistent with the use of materials available within an oasis agro-pastoral economy.

The weakly positive ovalbumin response in sample 47-4 raises the possibility that an egg-white-containing material was incorporated into the earthen plaster. Proteinaceous additives may influence wet adhesion, dry cohesion, and surface strength in earthen preparations, and egg-based materials have been documented in wall paintings and related clay-based artistic technologies [46,70], including the Bamiyan Buddhas [13,15]. Such use is therefore technologically plausible. However, the response was weak and was not reproduced in sample 47-5. Ageing, low concentration, heterogeneous distribution, and interactions with mineral components may all influence immunological detection in cultural heritage samples [24]. Accordingly, the result should be regarded as preliminary evidence for an ovalbumin-containing material rather than proof that egg white was systematically used as a binder or additive throughout Cave No. 47 of the Kizil Grottoes.

Above the earthen plaster, Raman spectroscopy identified anhydrite as the principal crystalline phase currently preserved in the white ground layer. Previous studies of Kizil wall paintings have reported both gypsum and anhydrite in calcium sulfate-based ground layers [18,19,20]. These reports indicate that calcium sulfate-based ground materials were used at Kizil, with both gypsum and anhydrite occurring in different samples. The white calcium sulfate layer would have provided a relatively smooth and light-coloured intermediate surface between the earthen plaster and the overlying paint layers [14,52], but the identification of anhydrite does not establish the original raw material or preparation route. The ground could have involved natural anhydrite associated with gypsum, thermally treated gypsum, or a calcium sulfate whose hydration state changed after application. The overlying red ground contained both hematite and anhydrite, and SEM–EDX shows Fe enrichment within the red layer together with broadly overlapping Ca and S distributions across the ground layer system. These red layer is therefore better interpreted as a hematite-bearing, anhydrite-rich preparation than as a pure iron oxide layer. Similar combinations of iron oxide colorants and calcium sulfate materials have been reported at the Zhongshan Grottoes (Shaanxi, China) [71], in the Turpan region [72], and elsewhere at the Kizil Grottoes [19], supporting the technological plausibility of such a formulation. The irregular, locally interwoven interface between the white and red grounds may reflect application onto a still-damp substrate or limited mechanical interpenetration, but it does not conclusively demonstrate that hematite was premixed with the calcium sulfate before application. The shared anhydrite component nevertheless suggests technological continuity between the successive white and red grounds. More broadly, the white and red layers appear to form a compositionally related calcium sulfate-based ground layer system, with the red layer distinguished primarily by the addition of hematite. In some areas, the white-red ground layer sequence is locally duplicated (Figure 3a). Such repetition may reflect renewed surface preparation associated with repair or redecoration. However, because no continuous intervening paint layer or clearly weathered interface was identified between the two white–red couplets, repeated application during a single decorative campaign cannot be excluded. The duplicated sequence therefore indicates more than one episode of ground application, but cannot at present be assigned unequivocally to a separate redecoration phase.

**Figure 8:**
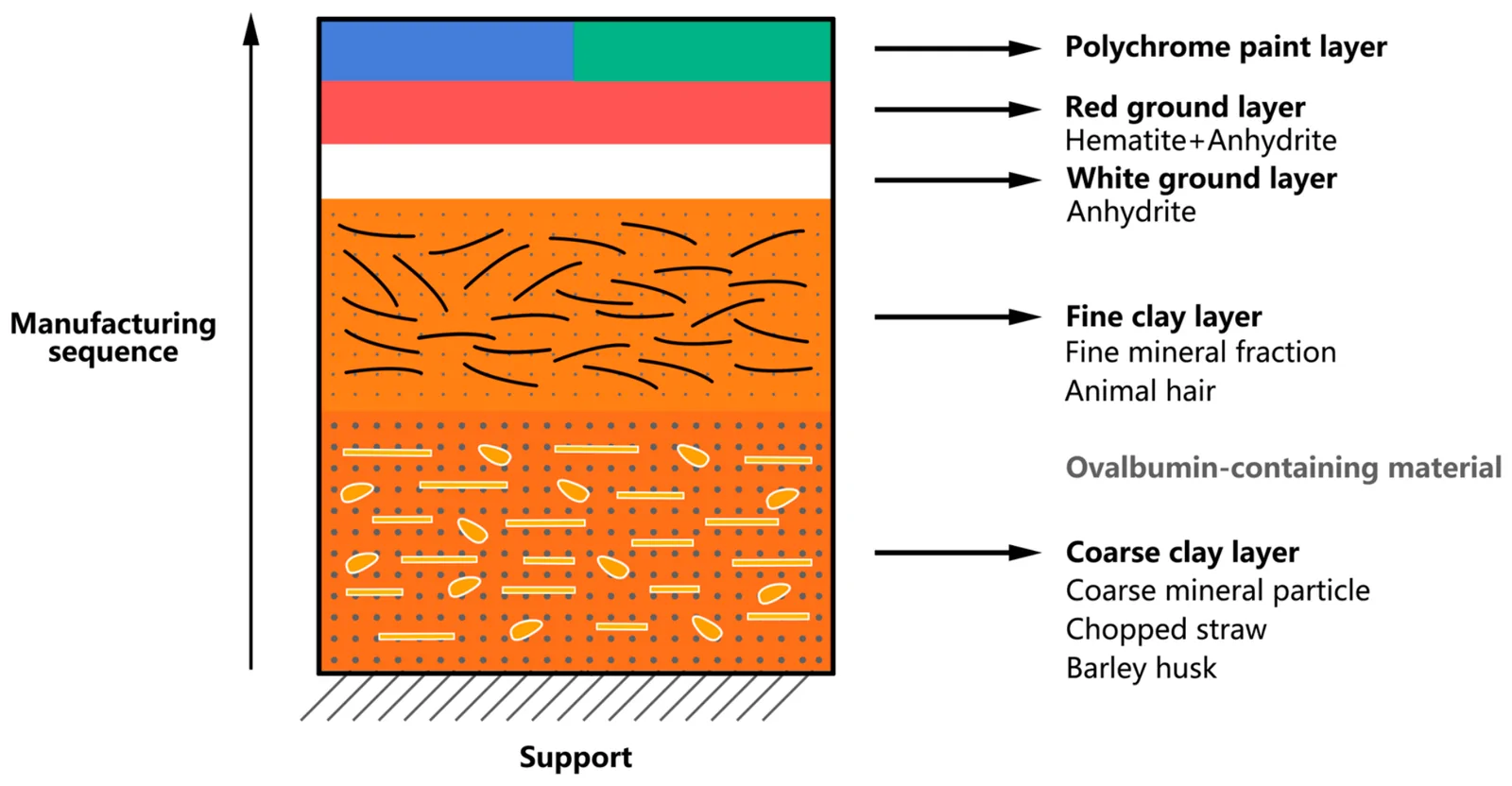
Schematic reconstruction of the inferred manufacturing sequence.

Taken together, these results allow the principal manufacturing sequence of the earthen plaster and ground layer to be reconstructed (Figure 8). A relatively coarse earthen mixture containing coarse mineral particles, chopped straw, and barley husks was first applied to form the lower coarse clay layer. This was followed by a finer mineral mixture incorporating animal hair, producing a more homogeneous fine clay layer and completing the earthen plaster. The prepared earthen surface was then covered by an anhydrite-dominated white ground layer, followed by a hematite-bearing, anhydrite-rich red ground layer and, finally, the polychrome paint layer. A weakly positive ovalbumin response from sample 47-4 provides preliminary evidence for the presence of an ovalbumin-containing material within the earthen plaster. The significance of this sequence lies not simply in the use of coarse and fine clay layers, a widespread feature of earthen plaster technologies, but in the coordinated differentiation of mineral fractions and organic inclusions between successive layers. Coarse plant-derived inclusions were concentrated in the lower layer, whereas animal hair was preferentially incorporated into the finer upper clay layer. Combined morphological and spectroscopic evidence is compatible with a provisional attribution of the hair to cattle, although species-level identification remains uncertain. Together with the tentative detection of an ovalbumin-containing material, these findings indicate that Kucha craftsmen combined locally available agricultural by-products and animal-derived materials within a deliberately stratified earthen plaster and ground layer technology.

## 5. Conclusions

This study provides an integrated characterization of the earthen plaster and ground layer system associated with the Nirvana platform in Cave No. 47 of the Kizil Grottoes. The combined stratigraphic, particle-size, mineralogical, spectroscopic, immunological, and archaeobotanical evidence establishes a representative manufacturing sequence comprising an underlying support, a relatively thick coarse clay layer, a thinner fine clay layer, an anhydrite-dominated white ground layer, a hematite-bearing, anhydrite-rich red ground layer, and the overlying polychrome paint layer. The coarse and fine clay layers together constitute the earthen plaster, whereas the white and red layers form a compositionally related calcium sulfate-based ground layer.

The two earthen layers were differentiated in particle-size distribution, crystalline mineral composition, and fibrous inclusions. The coarse clay layer displayed a broader particle-size distribution, a substantially larger coarse-particle fraction, and abundant chopped straw and barley husks. The fine clay layer was more homogeneous, contained fewer coarse particles, and was reinforced with numerous slender animal hairs. Morphological and spectroscopic observations support identification of these fibres as mammalian hair and are compatible with a provisional attribution to cattle, although species-level identification remains uncertain. A weakly positive ovalbumin response further provides preliminary evidence for an ovalbumin-containing material within the earthen plaster, but its precise technological role cannot yet be established.

The ground layers show a similarly differentiated material strategy. The white ground is dominated by anhydrite, whereas the overlying red ground contains hematite together with anhydrite. Their shared calcium sulfate component suggests technological continuity between successive ground applications, while the incorporation of hematite is consistent with the comparatively pale red appearance of the upper layer. Taken together, the results indicate that mineral fractions, plant-derived inclusions, animal hair, and calcium sulfate-based preparation materials were distributed differently across successive layers, reflecting a deliberately stratified technological system. Methodologically, this study demonstrates the value of integrating stratigraphic observation with granulometric, mineralogical, spectroscopic, immunological, and archaeobotanical analyses for reconstructing complex earthen plaster and ground layer technologies.

The manufacturing sequence documented at the Nirvana platform of Cave No. 47 conforms broadly to the multilayer earthen plaster traditions known across Asian Buddhist sites, while its distinctive combination of mineral, agricultural, and animal-derived materials reflects a local technological adaptation within ancient Kucha. These findings contribute to the understanding of monumental Buddhist image-making along the Silk Roads and establish an important material basis for the future conservation of the surviving Nirvana platform remains. In particular, the demonstrated compositional differentiation between successive layers highlights the need to consider stratigraphic heterogeneity when assessing conservation requirements. Comparative investigation of additional Kizil caves and related sites will be necessary to determine how widely this technological system was employed and how it developed across the region.

## Figures and Tables

**Table 1:** Basic information of the investigated samples.

Sample No.	Sampling Location	Sample Description
47-1	Upper halo area of the Nirvana platform in the rear chamber, to the left of the Buddha’s face	White ground and paint layers
47-2	Upper halo area of the Nirvana platform in the rear chamber	A hair-bearing tissue fragment from the fine clay layer
47-3	Upper halo area of the Nirvana platform in the rear chamber	Fine clay layer
47-4	Upper halo area of the Nirvana platform in the rear chamber	Earthen plaster (Fine and coarse layers)
47-5	Right wall of the rear chamber	Earthen plaster (Fine and coarse layers)
47-6	Found on the Nirvana platform	Detached fragment comprising multiple layers

**Table 2:** Indirect ELISA screening results for samples 47-4 and 47-5.

Sample No.	Target	Replicate Absorbance Values	Mean Absorbance	LOD	Positive Control Mean	RI (%)	Result
47-4	Ovalbumin	0.082, 0.073, 0.086	0.0803	0.0539	1.809	1.51	Weakly positive
47-4	Casein	0.031, 0.026, 0.029	0.0287	0.1315	1.319	−8.66	Negative
47-4	type I Collagen	0.052, 0.055, 0.055	0.0540	0.0669	1.636	−0.82	Negative
47-5	Ovalbumin	0.040, 0.038, 0.043	0.0403	0.0539	1.809	−0.77	Negative
47-5	Casein	0.032, 0.031, 0.037	0.0333	0.1315	1.319	−8.26	Negative
47-5	type I Collagen	0.051, 0.048, 0.049	0.0493	0.0669	1.636	−1.12	Negative

Reaction intensity (RI) was calculated as RI = (Asample − LOD)/(Apositive control − LOD) × 100%. The reaction intensity was classified as follows: RI ≤ 0%, negative; 0 < RI ≤ 10%, weakly positive; 10 < RI ≤ 30%, positive; and RI > 30%, strongly positive. Negative RI values indicate sample responses below the assay-specific LOD.

**Table 3:** Particle-size parameters of the fine and coarse clay layers.

Parameter	Fine Clay Layer	Coarse Clay Layer
D[4,3] (μm)	14.291 ± 0.131	39.407 ± 2.170
D[3,2] (μm)	4.744 ± 0.035	7.014 ± 0.161
d(0.1) (μm)	1.872 ± 0.009	2.509 ± 0.049
d(0.5) (μm)	8.157 ± 0.096	15.817 ± 0.482
d(0.9) (μm)	33.415 ± 0.601	116.197 ± 10.287
Span	3.866 ± 0.027	7.179 ± 0.434
Specific surface area (m^2^/g)	1.263 ± 0.012	0.856 ± 0.020

## Data Availability

Data are contained within the article.
